# The association of depressive symptoms, personality traits, and sociodemographic factors with health-related quality of life and quality of life in patients with advanced-stage lung cancer: an observational multi-center cohort study

**DOI:** 10.1186/s12885-020-06823-3

**Published:** 2020-05-18

**Authors:** Mark de Mol, Sabine Visser, Joachim Aerts, Paul Lodder, Nico van Walree, Huub Belderbos, Brenda den Oudsten

**Affiliations:** 1 Department of Pulmonary Diseases, Amphia Hospital, P.O. Box 90158, 4800 RK Breda, The Netherlands; 2 Department of Pulmonary Diseases, Erasmus MC Cancer Institute, P.O. Box 2040, 3000 CA Rotterdam, The Netherlands; 3 Department of Epidemiology, Erasmus MC – University Medical Centre Rotterdam, P.O. Box 2040, 3000 CA Rotterdam, The Netherlands; 4 Department of Methodology and Statistics, Tilburg University, P.O. Box 90151, 5000 LE Tilburg, The Netherlands; 5 Department of Medical and Clinical Psychology, Centre of Research on Psychological and Somatic Disorders (CoRPS), Tilburg University, P.O. Box 90151, 5000 LE Tilburg, The Netherlands

**Keywords:** Cancer, Depression, Lung neoplasms, Oncology, Personality traits, Quality of life

## Abstract

**Background:**

Identification of patient-related factors associated with Health-Related Quality of Life (HRQoL) and Quality of Life (QoL) at the start of treatment may identify patients who are prone to a decrease in HRQoL and/or QoL resulting from chemotherapy. Identification of these factors may offer opportunities to enhance patient care during treatment by adapting communication strategies and directing medical and psychological interventions. The aim was to examine the association of sociodemographic factors, personality traits, and depressive symptoms with HRQoL and QoL in patients with advanced-stage lung cancer at the start of chemotherapy.

**Methods:**

Patients (*n* = 151) completed the State-Trait Anxiety Inventory (trait anxiety subscale), the Neuroticism-Extraversion-Openness-Five Factor Inventory (NEO-FFI), the Center for Epidemiologic Studies Depression (CES-D), the World Health Organization Quality of Life-BREF (WHOQOL-BREF), and the European Organisation for Research and Treatment of Cancer Quality of Life Questionnaire-Core 30 (EORTC QLQ-C30). Simple linear regression analyses were performed to select HRQoL and QoL associated factors (a *P* ≤ 0.10 was used to prevent non-identification of important factors) followed by multiple linear regression analyses (*P* ≤ 0.05).

**Results:**

In the multiple regression analyses, CES-D score (β = − 0.63 to − 0.53; *P*-values < 0.001) was most often associated with the WHOQOL-BREF domains and general facet, whereas CES-D score (β = − 0.67 to − 0.40; *P*-values < 0.001) and Eastern Cooperative Oncology Group (ECOG) performance status (β = − 0.30 to − 0.30; *P*-values < 0.001) were most often associated with the scales of the EORTC QLQ-C30. Personality traits were not related with HRQoL or QoL except for trait anxiety (Role functioning: β = 0.30; *P* = 0.02, Environment: β = − 0.39; *P* = 0.007) and conscientiousness (Physical health: β = 0.20; *P*-value < 0.04).

**Conclusions:**

Higher scores on depressive symptoms and ECOG performance status were related to lower HRQoL and QoL in patients with advanced-stage non-small cell lung cancer. Supportive care interventions aimed at improvement of depressive symptoms and performance score may facilitate an increase of HRQoL and/or QoL during treatment.

## Background

Patients with advanced-stage lung cancer have a poor prognosis [1]. A 5 year survival of 6% was reported in patients with stage IV non-small cell lung cancer according to the datasets of the International Association for the Study of Lung Cancer staging project [1]. In addition, treatment may be associated with considerable side effects, which can directly influence Health-Related Quality of Life (HRQoL) [2] or even QoL in patients with metastatic cancer. Therefore, treatment goals should not be solely focused on survival benefits, but also consider the effect on patients’ HRQoL and QoL.

HRQoL focusses on health and represents the impact of disease and treatment on the feelings patients have about their functional capabilities and well-being [3]. QoL assesses patients’ feelings (i.e., satisfied or bothered) about their functioning and well-being in at least three key areas (i.e., physical, psychological and social well-being). It also evaluates a patient’s feelings related to their environment (e.g., satisfaction with living conditions) or spirituality (e.g., meaningfulness of personal life). A recent study underscores the additional value of spirituality for a patient’s well-being as it observed that better cognitive and emotional functioning was seen in cancer patients with higher spiritual well-being [4]. Patients with better global Health Status/QoL also had higher spiritual well-being. In addition, besides the additional assessment of a patient’s environment and spirituality, a QoL instrument also contains positively phrased items.

In studies that investigate new therapies in lung cancer, often HRQoL is evaluated and not QoL. These studies evaluate HRQoL to determine the impact of treatment on cancer patients’ well-being. QoL may be used in a similar manner and provides further information as it enables a more comprehensive assessment of a patients well-being than HRQoL. In a clinical setting, application of HRQoL and QoL questionnaires may be used to identify aspects of a patient’s health he/she is bothered with. For instance, it may be used to monitor the effects of treatment on a patient’s well-being. Moreover, HRQoL and QoL assessment may provide opportunities to apply interventions to improve HRQoL and QoL. Regarding the questionnaires to evaluate HRQoL and QoL in lung cancer: according to the definition of the WHO, no lung cancer specific QoL questionnaire has been developed. Some questionnaires are specifically developed for lung cancer (e.g., European Organization for Research and Treatment of Cancer Quality of Life Questionnaire Core 30 (EORTC QLQ-C30), Functional assessment of Cancer Therapy-Lung), although they are considered as a HRQoL instrument or even a Health Status questionnaire in case of the EORTC QLQ-C30 given the emphasis on physical complaints rather than well-being.

Several factors have been associated with HRQoL in patients with lung cancer (i.e., age, performance status, gender, education, and having a spouse/partner [5–7]) in the past decades. In addition, in patients with cancer, depressive symptoms are negatively related with HRQoL [8, 9]. However, given that depressive symptoms also have been negatively associated with spiritual well-being [4], investigating the association between depressive symptoms and QoL may provide further information about the relation between depressive symptoms and a patient’s well-being.

Personality has been associated with depressive symptoms in chronic illnesses [10, 11] and reduced emotional HRQoL in heart failure patients [12]. In breast cancer, high scores on certain personality traits (i.e., trait anxiety and neuroticism) were associated with lower overall QoL scores over time [13]. Considering these results, the assessment of the association of personality traits with HRQoL and QoL at the start of treatment in patients with lung cancer may help identify patients who are prone to low levels of HRQoL and/or QoL. Moreover, taking knowledge of patient’s personality traits may be of importance as they are linked with coping mechanisms. It may help personalize communication strategies and the manner in which supportive care is delivered. This may be of importance to increase, for instance, treatment adherence.

However, studies that have investigated the relation between the above mentioned factors (i.e., personality, sociodemographic, clinical and psychological factors (e.g., depressive symptoms)) and HRQoL and/or QoL in patients with lung cancer are not reported. This is unfortunate since lung cancer patients are at risk to have lower scores on functioning and well-being given their disease, treatment-related adverse events, and life expectancy [14]. Moreover, a study by Temel and colleagues demonstrated that early palliative care in newly diagnosed lung cancer patients improved HRQoL and depressive symptoms at 12 and 24 weeks after treatment commenced [15]. Therefore, knowledge of which factors are associated with HRQoL and QoL prior to or at the start of treatment may be worthwhile, because these factors may require additional care in individual patients during treatment.

Contemplating on these considerations, we aimed to evaluate to which extent depressive symptoms and personality traits solely and among variables related with HRQoL (i.e., age, performance status, gender, education, and having a spouse/partner [5–7]) are associated with HRQoL and QoL in patients with advanced-stage lung cancer prior to or at the start of treatment. We expected depressive symptoms to be associated with lower scores on HRQoL [8, 9] and QoL. In addition, we estimated neuroticism and trait anxiety to be associated with decreased HRQoL and QoL scores [13].

## Methods

### Study population

PERSONAL is a prospective observational multi-center cohort study of patients with stage IIIB or IV non-squamous non-small cell lung cancer and unresectable mesothelioma receiving pemetrexed. The present study is part of PERSONAL. PERSONAL aims to study the pharmacokinetic and pharmacologic effects of pemetrexed. In addition, patient reported outcomes are measured. Patients were recruited from October 2012 to November 2014 from three teaching hospitals (Erasmus University Medical Center, Amphia Hospital and Sint Franciscus Gasthuis hospital) and a regional hospital (Bravis hospital). Patients were enrolled if they met the following criteria: they were aged 18 years or older, had a cytological or histological confirmed diagnosis of stage IIIB or IV non-squamous non-small cell lung cancer or unresectable malignant pleural mesothelioma, and started treatment with pemetrexed in combination with cisplatin or carboplatin as either first line or with pemetrexed monotherapy as second line. Patients were excluded if they were not able to read Dutch or could not complete the questionnaires because of a physical or mental condition. Eligibility was checked by two physicians dedicated to the project. Informed consent was obtained from all individual participants included in the study. All procedures were in accordance with the ethical standards of the institutional review board of the Erasmus University Medical Center in Rotterdam, The Netherlands (approval number MEC-2012-232) and with the 1964 Helsinki declaration and its later amendments or comparable ethical standards.

### Procedures

All questionnaires were administered during consultations or by mail and completed after diagnosis and just before or at the first day of the first cycle of chemotherapy. Patients were asked once to complete the questionnaires and not repeatedly to prevent that they could feel obliged to comply to the researchers’ request. In addition, we collected sociodemographic information (i.e., age, gender, educational level, ethnicity, employment, partner status) and clinical information (i.e., cancer stage, type of tumour, line of therapy, and the Eastern Cooperative Oncology Group (ECOG) performance status) from the hospital electronic information records and during regular consultations.

### Study measures

#### Quality of life

The World Health Organization Quality of Life-BREF questionnaire (WHOQOL-BREF) is a cross-cultural and generic QoL instrument [16]. The WHOQOL-BREF comprises 24 items divided over four domains plus two general facet items describing overall QoL and general health. Items are scored on a Likert-scale from one (worst QoL) to five (best QoL). The domains represent physical health (seven items), psychological health (six items), social relationships (three items) and environment (eight items). Examples of items are: How satisfied are you with your capacity for work? (physical health); How safe do you feel in your daily life? (psychological health); How satisfied are you with your personal relationships? (social relationships); How satisfied are you with your transport? (environment). WHOQOL-BREF domains are scored on a 4–20 scale and the general facet on a 2–10 scale with higher scores indicating better QoL [16, 17]. The WHOQOL-BREF has satisfactory psychometric properties in patients with lung cancer [18], chronic diseases and other cancer types [16], except for the social relationships domain (i.e., relatively low Cronbach’s alpha < 0.70).

#### Health-related quality of life

The European Organization for Research and Treatment of Cancer-Quality of Life Questionnaire-Core 30 (EORTC-QLQ-C30) is a cancer specific HRQoL instrument originally developed in patients with lung cancer [19]. It consists of 30 items and incorporates a global Health Status/QoL scale, five functional scales and 13 items assessing symptoms or problems. The functional scales represent physical functioning (five items), cognitive functioning (two items), emotional functioning (four items), role functioning (two items), and social functioning (two items). Examples of items are: Do you have trouble taking a long walk? (physical functioning); Have you had difficulty remembering things? (cognitive functioning); Did you feel depressed? (emotional functioning); Has your physical condition or medical treatment interfered with your family life? (role functioning); Has your physical condition or medical treatment interfered with your social activities? (social functioning). EORTC QLQ-C30 domains are scored on a 0–100 scale, with higher scores on the functional scales being indicative of better HRQoL, whereas higher scores on the symptom scales represent worse symptoms [19]. The EORTC has demonstrated acceptable psychometric properties [20].

#### Personality traits

The State-Trait Anxiety Inventory (STAI) questionnaire assesses state and trait anxiety [21]. We used the 10-item STAI trait anxiety subscale (short version), which was developed in women suspected with breast cancer and breast cancer survivors [22]. Trait anxiety refers to the tendency to respond to threatening situations with increased anxiety intensity [13]. It is considered to be a personality factor. Items are scored on a four-point scale ranging from one (almost never) to four (almost always). An example of an item is: I worry too much over something that really doesn’t matter. A score of ≥ 22 is indicative for high trait anxiety [22]. The original Dutch translation of the STAI [21, 23] and the 10-item subscale itself [22] have good psychometric properties.

The 60-item Neuroticism-Extraversion-Openness-Five Factor Inventory questionnaire (NEO-FFI) assesses personality based on the Five Factor Model [24–26]. It describes neuroticism, extraversion, openness to experience, agreeableness, and conscientiousness. Neuroticism measures emotional stability. Extraversion assesses the level to which orientation, energy and attention are focused on the outside world instead of the inner world. Openness reflects to an open attitude towards experiences, beliefs and, people. Agreeableness relates to a person’s level of being empathic, cooperative, and considerate. Conscientiousness refers to the level of being careful, diligent, and orderly. Items are scored on a five-point scale with scores ranging from one (totally disagree) to five (totally agree). Examples of items are: I often feel inferior to others (neuroticism); I laugh easily (extraversion); Once I find the right way to do something, I stick to it (openness); I try to be courteous to everyone I meet (agreeableness); I keep my belongings clean and neat (conscientiousness). The NEO-FFI has good psychometric properties in patients with multiple sclerosis [27] and has been used in patients with cancer [28, 29]. For this study the raw scores of the NEO-FFI domains were used.

#### Depressive symptoms

The Center for Epidemiologic Studies Depression Scale (CES-D) is a 20-item questionnaire which evaluates depressive symptoms [30]. We used the 16-item version of the CES-D, in which the four positively formulated items of the original CES-D are removed [31, 32] since they lacked validity and did not correspond well with the definition of depressive symptoms. Items are scored on a four-point scale with scores ranging from zero (rarely) to three (mostly). An example of an item is: I felt that people dislike me. The CES-D has good psychometric properties in cancer patients [31, 33, 34]***.***

### Statistics

Patient characteristics between patients who completed the questionnaires and those who did not were compared with Fisher’s exact test and the independent T-test.

Given the sample size of 151 patients, simple linear regression analyses were performed as a minimal sample size of 50 + 8 m (in which m is the number of predictors) is recommended [35]. Analyses were conducted for sociodemographic variables (i.e., age, gender, ethnicity, education, employment, partner status), ECOG performance status, CES-D score, STAI Trait subscale score, and NEO-FFI subscale scores to identify possible factors associated with the WHOQOL-BREF domains and EORTC QLQ-C30 scales. To prevent non-identification of important variables by using a more strict alpha of ≤ 0.05, variables with an alpha of ≤ 0.10 were selected as possible predictors [36, 37].

With the variables associated with the WHOQOL-BREF domains and EORTC QLQ-C30 scales according to the simple linear regression analyses, multiple linear regression analyses were performed. An alpha of ≤ 0.05 was used to identify significant factors in the multiple linear regression analyses.

To contribute to statistical power several actions were taken. Firstly, a priori hypotheses were formulated according to the literature that we aimed to test in a homogenous patient population to minimise variability in the outcome measure of interest. Secondly, a recommended rule of thumb was used to calculate sample size [35] and patients were encouraged by the investigators to complete questionnaires to minimise the number of dropouts. Lastly, to minimise the risk for a type I error we applied Benjamini-Hochberg correction to adjust for multiple analyses.

Furthermore, to confirm that the results of our multivariable analyses were supported by sufficient statistical power, we performed a post-hoc power-analysis. Given an alpha of 0.05, a total of no more than nine factors for each multivariable model, and 151 patients, we were able to find an effect size (i.e., partial R^2^) of 3.98%. This means that the analyses were sufficiently powered to detect factors able to explain at least 3.98% variation in a HRQoL/QoL domain/scale score.

All analyses were performed using IBM SPSS Statistics for Windows version 21.0.

## Results

### Patient characteristics

**Fig. 1:**
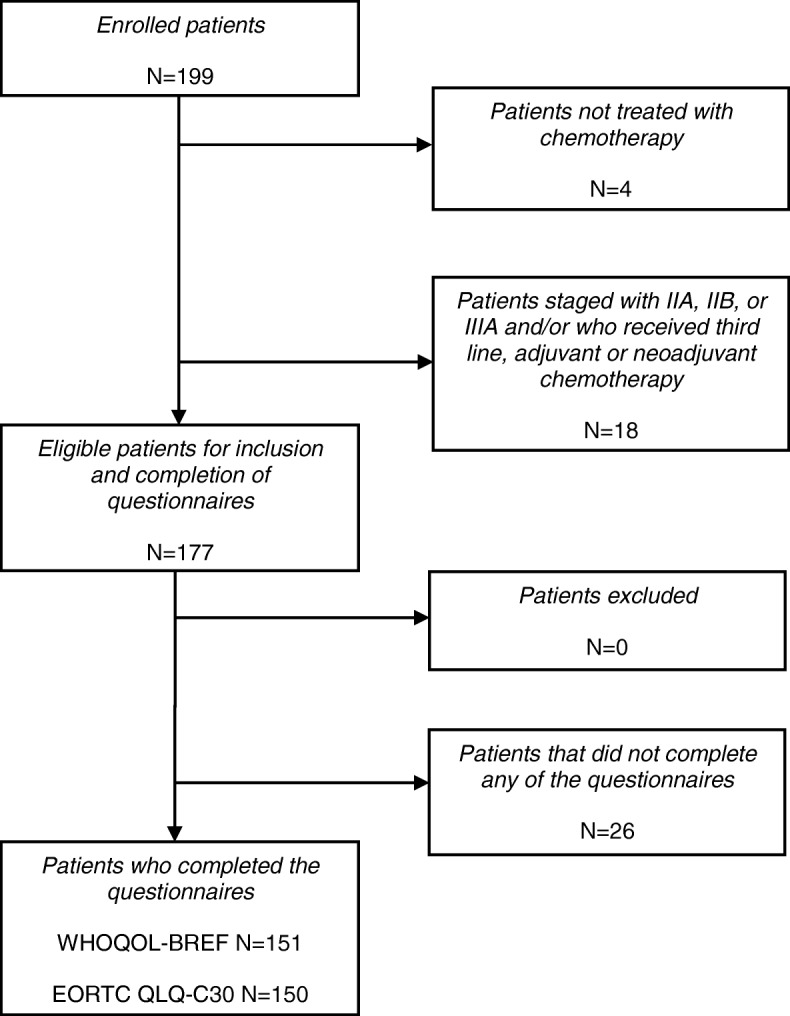
Selection of patients. Abbreviations: N, number of patients; WHOQOL-BREF, World Health Organization Quality of Life-BREF questionnaire; EORTC QLQ-C30, European Organization for Research and Treatment of Cancer Quality of Life Questionnaire Core 30

Figure 1 demonstrates the selection of patients. In total, 151 patients were used for analyses with the WHOQOL-BREF and 150 patients for analyses with the EORTC QLQ-C30. 89% of patients completed all domains of all questionnaires. Table 1 summarizes the patient characteristics of the included patients and the 26 patients who did not complete any of the questionnaires. In general, reasons for non-completion of questionnaires were related to the stress patients experienced resulting from a diagnosis of advanced-stage lung cancer, the near start of chemotherapy, and a poor prognosis. These patients did not differ from the 151 included patients according to the age, gender, ethnicity, employment, partner status, cancer stage, tumour type, and line of therapy, except for performance status. The proportion of patients with a performance status of two or higher was larger in the patients that were not available for the analyses than the included patients. WHOQOL-BREF domain scores, EORTC QLQ-C30 scale score, personality scale scores and CES-D scores are summarized in Table 2.

**Table 1:** Characteristics of study population

Characteristic	Patients who completed questionnaires (***N*** = 151)	Patients who did not complete any questionnaire (***N*** = 26)	P_**a**_
Age, years_b_			
Mean (SD)	63.3 (9.1)	63.7 (8.7)	0.85
Min, max	37, 83	47, 80	
Gender			
Male	82 (54.3)	12 (46.2)	0.53
Ethnicity			
White / Caucasian	142 (94.0)	25 (96.2)	1.00
Other	9 (6.0)	1 (3.8)	
Education_c_			
Low	113 (74.8)		
High	32 (21.2)		
Unknown	1 (0.7)	26 (100.0)	
Employment_b_			
Yes	38 (25.2)	1 (3.8)	0.26
No	112 (74.2)		
Unknown	1 (0.7)	25 (96.2)	
Partner status_b_			
Partner	122 (80.8)	1 (3.8)	1.00
No partner	28 (18.5)		
Unknown	1 (0.7)	25 (96.2)	
Cancer stage_b_			
Locally advanced (IIIB)	19 (12.6)	2 (7.7)	0.76
Metastatic (IV)	124 (82.1)	23 (88.5)	
Other	8 (5.3)	1 (3.8)	
Type of tumor_b_			
Adenocarcinoma	136 (90.1)	24 (92.3)	1.00
Large cell carcinoma, mesothelioma, other	15 (9.9)	2 (7.7)	
Line of therapy			
irst	140 (92.7)	22 (84.6)	0.24
econd	11 (7.3)	4 (15.4)	
ECOG performance status_b_			
Grade 0 or 1	135 (89.4)	18 (69.2)	0.02
Grade 2 or higher	14 (9.3)	7 (26.9)	
Unknown	2 (1.3)	1 (3.8)	

Values are given in numbers (percentages) unless stated otherwise. _a_P-values reflect differences between patients who completed any questionnaire and those who did not

_b_Measured at the start of treatment with chemotherapy

_c_Low education: persons whose highest level of education is primary education, lower general education or lower vocational education. High education: persons whose highest level of education is higher general education, higher vocational education or university.

*Abbreviations*: *N* number of patients, *SD* standard deviation, *ECOG* Eastern Cooperative Oncology Group (ECOG)

**Table 2:** WHOQOL-BREF, EORT QLQ-C30, NEO-FFI, CES-D, and STAI trait scale/domain scores

Questionnaire	Scale/domain	***N***	Median	Mean (SD)	Min, max (IQR)	Range
WHOQOL-BREF	Physical health	145	13.1	12.9 (3.1)	4.0, 20.0 (4.6)	16
	Psychological health	145	14.7	14.5 (2.4)	9.3, 20.0 (3.3)	10.7
	Social relationships	145	16.0	16.3 (2.5)	8.0, 20.0 (3.3)	12
	Environment	145	16.0	15.9 (2.2)	10.0, 20.0 (3.0)	10
	General facet	142	6.0	5.8 (1.7)	2.0, 10.0 (2.0)	8
EORTC QLQ-C30	Physical functioning	150	66.7	68.1 (24.1)	6.7, 100.0 (33.3)	93.3
	Cognitive functioning	142	83.3	80.3 (23.1)	0.0, 100.0 (33.3)	100
	Emotional functioning	142	75.0	67.3 (24.0)	0.0, 100.0 (33.3)	100
	Role functioning	149	66.7	55.1 (32.8)	0.0, 100.0 (50.0)	100
	Social functioning	142	83.3	71.5 (27.0)	0.0, 100.0 (50.0)	100
	Global Health Status/QoL	142	58.3	54.8 (25.5)	0.0, 100.0 (41.7)	100
NEO-FFI	Neuroticism	137	28.0	28.1 (7.4)	12.0, 53.0 (8.5)	41
	Extraversion	133	40.0	40.4 (6.6)	22.0, 56.0 (9.5)	34
	Openness	134	34.0	34.3 (5.9)	20.0, 50.0 (7.3)	30
	Agreeableness	139	43.0	42.8 (5.0)	29.0, 54.0 (6.0)	25
	Conscientiousness	134	47.0	47.1 (5.7)	34.0, 60.0 (9.3)	26
STAI	Trait anxiety	147	17.0	17.7 (5.3)	10.0, 34.0 (8.0)	24

*Abbreviations*: *WHOQOL-BREF* World Health Organization Quality of Life-BREF questionnaire, *EORTC QLQ-C30* European Organization for Research and Treatment of Cancer Quality of Life Questionnaire Core 30, *NEO-FFI*, Neuroticism-Extraversion-Openness-Five Factor Inventory, *CES-D* Center for Epidemiologic Studies Depression Scale, *STAI* State-Trait Anxiety Inventory, *N* number of patients, *SD* standard deviation, *IQR* inter quartile range

### Linear regression analyses

Results of the simple linear regression analyses for each of the HRQoL and QoL domains/scales are demonstrated in Table 3 (see also Online Resource 1). Table 4 demonstrates the multiple linear regression analyses for the WHOQOL-BREF domains and general facet. After Benjamini-Hochberg correction, CES-D score was negatively associated with the general facet and with the physical and psychological health domains. For the EORTC QLQ-C30 scale scores, CES-D score was negatively associated with the functioning scales and the global Health Status/QoL score (Table 5). All of the standardized betas for the significant associations between CES-D score and the domains/scales of the WHOQOL-BREF and EORTC QLQ-C30 were larger than 0.40. After Benjamini-Hochberg correction, ECOG performance status was negatively associated with the physical and role functioning scale scores of the EORTC QLQ-C30 and with the physical health domain of the WHOQOL-BREF. For the NEO-FFI personality traits, only a positive association between the conscientiousness scale and the physical health domain of the WHOQOL-BREF was observed. Trait anxiety was negatively associated with environment (WHOQOL-BREF) and positively with role functioning (EORTC QLQ-C30). For the WHOQOL-BREF explained variances ranged from 0.20 to 0.55 and for the EORTC QLQ-C30 from 0.36 to 0.66.

**Table 3:** Results of the univariable regression analyses with the WHOQOL-BREF and EORTC QLQ-C30 domains/scales as dependent variables

	WHOQOL-BREF					EORTC QLQ-C30					
	General facet	Physical health	Psychological health	Social relationships	Environment	General Health Status/QoL	Physical functioning	Role functioning	Emotional functioning	Cognitive functioning	Social functioning
**Independent variables**	β	β	β	β	β	β	β	β	β	β	β
Age	− 0.168**	0.042	0.032	0.074	0.069	− 0.052	− 0.002	0.045	− 0.074	0.058	0.120
Gender_a_	−0.011	−0.121	− 0.075	0.185**	0.077	−0.114	− 0.218**	− 0.185**	− 0.039	− 0.163*	− 0.105
Marital status: no partner versus having a partner_b_	−0.078	− 0.101	0.039	0.170**	0.079	− 0.043	− 0.076	− 0.038	− 0.132	− 0.010	− 0.163*
Educational level: low versus high_c_	0.023	0.029	0.086	0.111	0.170**	−0.047	−0.006	− 0.008	0.046	0.214**	0.024
Ethnicity: Caucasian versus other ethnicity_d_	−0.127	−0.023	− 0.103	0.049	0.010	−0.056	− 0.001	−0.018	− 0.034	0.007	− 0.079
Employment: yes versus having no job_e_	0.218**	0.203**	0.096	−0.018	0.075	0.257**	0.246**	0.135*	0.120	0.148*	0.182**
ECOG: 0 to 1 versus 2 or higher_f_	−0.220**	− 0.301**	−0.197**	− 0.211**	−0.123	− 0.200**	−0.311**	− 0.319**	−0.127	0.012	−0.220**
CES-D	−0.534**	−0.575**	− 0.653**	−0.168**	− 0.465**	−0.589**	− 0.482**	−0.525**	− 0.786**	−0.575**	− 0.505**
STAI Trait	−0.236	− 0.356**	−0.518**	− 0.225**	−0.522**	− 0.260**	−0.209**	− 0.151*	−0.597**	− 0.341**	−0.280**
NEO-FFI neuroticism	−0.153*	−0.296**	− 0.494**	−0.230**	− 0.389**	−0.269**	− 0.163*	−0.217**	− 0.525**	−0.276**	− 0.231**
NEO-FFI extraversion	0.196**	0.210	0.278**	0.216**	0.209**	0.179**	0.179**	0.137	0.203**	0.016	0.195**
NEO-FFI openness	0.049	−0.132	0.002	0.039	−0.059	− 0.045	−0.114	− 0.155*	0.058	− 0.020	−0.067
NEO-FFI agreeableness	0.051	0.177**	0.163*	0.139	0.198**	0.153*	0.145*	0.135	0.240**	0.140	0.148*
NEO-FFI conscientiousness	0.172**	0.291**	0.314**	0.238**	0.295**	0.242**	0.243**	0.187**	0.181**	0.132	0.292**

*P-values of p ≤ 0.10

**P-values of p ≤ 0.05

_a_Male is reference

_b_No partner is reference

_c_Low educational level is reference

_d_Other ethnicity is reference

_e_No job is reference

_f_0 to 1 is reference

CES-D score, STAI trait score and NEO-FFI scale scores represent continuous variables

*Abbreviations*: *β* standardized beta**,***WHOQOL-BREF*, World Health Organization Quality of Life-BREF questionnaire, *EORTC QLQ-C30* European Organization for Research and Treatment of Cancer Quality of Life Questionnaire Core 30, *ECOG* Eastern Cooperative Oncology Group, *CES-D* Center for Epidemiologic Studies Depression Scale, *STAI* State Trait Anxiety Inventory, *NEO-FFI* Neuroticism-Extraversion-Openness Five-Factor Inventory

**Table 4:** Results of the multivariable regression analyses for the WHOQOL-BREF (*p* < 0.05)

Independent variables	N	B	SE	β	P-value	Corrected P-value_**a**_	95% CI for B	R^**2**^
	General facet							
Age	117	−0.041	0.015	−0.232	0.006	0.024	−0.070, − 0.012	0.402
CES-D		−0.133	0.021	−0.625	< 0.001	< 0.001	−0.175, − 0.091	
Physical health								
ECOG: 0 to 1 versus 2 or higher	117	−2.747	0.751	−0.262	< 0.001	< 0.001	−4.234, −1.259	0.517
CES-D		−0.221	0.035	−0.542	< 0.001	< 0.001	−0.291, − 0.151	
NEO-FFI conscientiousness		0.111	0.045	0.201	0.016	0.043	0.021, 0.200	
	Psychological health							
CES-D	117	−0.163	0.025	−0.534	< 0.001	0.000	−0.213, − 0.113	0.554
	Social relationships							
Gender	119	1.107	0.467	0.222	0.020	0.080	0.181, 2.032	0.204
Partner status: no partner versus having a partner		1.428	0.588	0.216	0.017	0.080	0.262, 2.594	
	Environment							
CES-D	116	−0.063	0.028	−0.224	0.026	0.091	−0.118, − 0.008	0.375
STAI Trait		−0.163	0.049	−0.392	0.001	0.007	−0.259, − 0.066	

_a_Benjamini-Hochberg method was used to correct P-values

*Abbreviations*: *WHOQOL-BREF* World Health Organization Quality of Life-BREF questionnaire, *N* number of patients, *B* unstandardized beta, *SE* standard error, *β* standardized beta, *CI* confidence interval, *R*^*2*^ explained varriance, *CES-D* Center for Epidemiologic Studies Depression Scale, *ECOG* Eastern Cooperative Oncology Group, *NEO-FFI* Neuroticism-Extraversion-Openness-Five Factor Inventory questionnaire, *STAI*, State Trait Anxiety Inventory

**Table 5:** Results of the multivariable regression analyses for the EORTC QLQ-C30 (p < 0.05)

Independent variables	N	B	SE	β	P-value	Corrected P-value_**a**_	95% CI for B	R^**2**^
	General Health Status/Quality of Life							
Employment: yes versus no job	116	10.405	4.358	0.183	0.019	0.076	1.764, 19.045	0.417
CES-D		−2.062	0.314	−0.627	< 0.001	< 0.001	−2.684, −1.439	
	Physical functioning							
Employment: no versus having a job	117	10.684	3.885	0.204	0.007	0.021	2.981, 18.386	0.453
ECOG: 0 to 1 versus 2 or higher		−23.586	5.958	−0.304	< 0.001	< 0.001	−35.398, −11.775	
CES-D		−1.357	0.284	−0.449	< 0.001	< 0.001	−1.921, − 0.793	
	Role functioning							
ECOG: 0 to 1 versus 2 or higher	120	−30.890	7.975	−0.299	< 0.001	< 0.001	−46.692, −15.088	0.414
CES-D		−2.197	0.384	−0.542	< 0.001	< 0.001	− 2.957, −1.437	
STAI Trait		1.840	0.687	0.295	0.009	0.024	0.479, 3.201	
	Emotional functioning							
CES-D	117	−2.044	0.222	−0.668	< 0.001	< 0.001	−2.483, −1.604	0.655
	Cognitive functioning							
Educational level: low versus high	129	9.344	4.060	0.170	0.023	0.069	1.307, 17.382	0.359
CES-D		−1.572	0.274	−0.536	< 0.001	< 0.001	−2.114, − 1.030	
	Social functioning							
Partner status: no partner versus having a partner	116	−12.786	5.817	−0.174	0.030	0.090	−24.318, −1.253	0.370
ECOG: 0 to 1 versus 2 or higher		−16.748	7.367	−0.188	0.025	0.090	−31.354, −2.141	
CES-D		−1.394	0.348	−0.401	< 0.001	< 0.001	−2.085, − 0.704	

_a_Benjamini-Hochberg method was used to correct P-values

*Abbreviations*: *EORTC QLQ-C30*, European Organization for Research and Treatment of Cancer Quality of Life Questionnaire Core 30, *N* number of patients, *B* unstandardized beta, *SE* standard error, *β* standardized beta, *CI* confidence interval, *R*^*2*^ explained varriance, *CES-D* Center for Epidemiologic Studies Depression Scale, *ECOG* Eastern Cooperative Oncology Group, *STAI* State Trait Anxiety Inventory

## Discussion

Due to a diagnosis of cancer and potential treatment-related side effects advanced-stage lung cancer patients are at risk to experience a decrease in HRQoL and QoL after they start with treatment. Physicians are aware of this [38] and try to optimize HRQoL and QoL. Evaluation of factors associated with HRQoL and QoL at the start of treatment may provide opportunities to prevent further deterioration of those areas of HRQoL and/or QoL that are related to these factors. To our knowledge, this prospective multi-centre observational study is the first that aimed to investigate if personality traits, depressive symptoms, and sociodemographic factors are associated with HRQoL and QoL in patients with advanced-stage lung cancer prior to or at the start of treatment. Considering that HRQoL merely reflects those components of QoL that are influenced by treatment and disease [3], we choose to include a QoL measure (i.e., WHOQOL-BREF) as well since this offers additional information describing patients’ feelings about their environment and spirituality/existentiality. We observed that higher levels of depressive symptoms were associated with decreased HRQoL and QoL except for social relationships and environment. Given the associations with both HRQoL and QoL and the fact that depressive symptoms are common [1, 2], our results emphasize the importance of physicians’ awareness for depressive symptoms in patients with advanced-stage lung cancer.

Compared to a recent study in Dutch patients with lung cancer, we observed a lower general health/QoL score (i.e., facet score of 7.0 (SD 1.4) versus 5.8 (SD 1.7) in this study) [39]. Probably this is due to the inclusion of solely patients with locally-advanced and metastatic lung cancer in our study whereas the referred study included patients with all stages of lung cancer with stage I and II comprising 45% of the study population. However, this difference in QoL underscores the need for the development of interventions to improve QoL in patients with advanced-stage lung cancer. In patients with breast and prostate cancer, it was reported that an easy-to-use well-being intervention (i.e., recording of positive experiences in a diary, listening to a mindfulness CD, planning a pleasurable activity) could positively influence overall QoL (i.e., facet score WHOQOL-BREF) [40]. Moreover, in a study with Iranian breast cancer patients an intervention of eight mindfulness group-based training sessions resulted in improved overall QoL and less depressive symptoms, anxiety, and stress compared to the control group [41]. Regarding HRQoL, a Cochrane review reported that exercise training resulted in improved global HRQoL although this was not observed for physical functioning. Also the risk of bias in all six included studies was high and the quality of evidence for the outcomes was low [42]. In another study, Nabilone, a synthetic cannabinoid used to improve caloric intake, resulted in improved aspects of HRQoL (i.e., role functioning, emotional functioning, and social functioning) [43]. Unfortunately, all of the mentioned studies are hampered by their design and relatively small sample sizes, although their results suggest that the development of interventions to improve HRQoL and QoL could be beneficial for patients with advanced-stage cancer. Therefore, randomized studies with larger patient populations are needed that could further develop and test the additional value of interventions designed to improve HRQoL and QoL. Such studies should particularly aim their proposed interventions at improving performance status and depressive symptoms as, according to our results, these factors contribute the most to HRQoL and QoL.

In the present study CES-D score was related to all HRQoL scales and QoL domains, except the WHOQOL-BREF domains social relationships and environment. Previousy, the CES-D score has been related with HRQoL and QoL in breast cancer [44, 45]. In the study by Hyphantis and colleagues, amongst others, age, stage of cancer, levels of anxiety, depressive symptoms, and use of repression were related with QoL [45]. In line with the results of the present study, they did not observe a relationship between social relationships and CES-D score. However, in another study in lung cancer patients significant depressive symptoms were associated with decreased QoL, including social relationships and environment [46]. Reasons for this may be related to differences in patient characteristics or the relatively large time since diagnosis (i.e., at least 20 months) that patients completed the questionnaires compared to our study. In our study, patients were at the start or prior to treatment whereas in the study by Gu et al. patients already received treatment for some time [46]. Treatment may have had an impact on the relation between depressive symptoms and QoL.

NEO-FFI personality traits were not associated with HRQoL and QoL in this study, except for conscientiousness. Trait anxiety was associated with only two HRQoL and QoL scales/domains, namely role functioning and environment. Considering that CES-D score was associated with almost all HRQoL and QoL scales/domains, we hypothesized whether the absent effect of personality traits on HRQoL and QoL was influenced by CES-D score. Therefore, new analyses were performed without CES-D score. For the WHOQOL-BREF, trait anxiety was associated with not only the environment domain, but also with physical and psychological health. Instead of an association with role functioning, trait anxiety was associated with the EORTC QLQ-C30 scales emotional functioning and social functioning. Previously, similar results have been observed. In a study with Turkish colorectal patients that received chemotherapy, patients with low trait anxiety (scale score < 45) had better HRQoL for all EORTC QLQ-C30 functioning scales and the global QoL/HS scale [47]. Another study in women under follow-up for breast cancer observed that the level of anxiety according to the total STAI score was related with the emotional functioning scale of the EORTC QLQ-C30 [48]. As such, our observations and the results of these studies emphasize the importance of trait anxiety as a factor associated with HRQoL and QoL, especially in the absence of depressive symptoms and may provide professionals opportunities to personalize the way they provide supportive care (e.g., by adapting communication strategies, stimulating effective coping mechanisms). Given that neuroticism has been linked with depressive symptoms in patients with lung cancer [49], we expected that the effect of neuroticism was masked by CES-D score. However, after removal of CES-D score from the models, neuroticism was not associated with any HRQoL scale or QoL domain. Furthermore, none of the other NEO-FFI personality traits were associated with HRQoL and QoL. In contrast, type D personality has previously been related with decreased HRQoL in patients with cancer [50, 51]. Given that in the past type D personality has been positively correlated with neuroticism and negatively with extraversion in healthy individuals [52, 53], it remains unclear why neuroticism or extraversion were not related with HRQoL or QoL in the present study. A reason for this may be that type D personality is more related with HRQoL and QoL than the NEO-FFI personality traits as was observed in female patients with ulcerative colitis [53]. Unfortunately, other studies that could further elucidate this lack of significance between NEO-FFI personality traits and HRQoL and QoL in cancer patients have not been reported. Therefore, the effect of personality traits according to the NEO-FFI on HRQoL and QoL remains unclear in patients with lung cancer.

We observed an unexpected result during the multiple regression analyses. First, the direction of the beta of the STAI trait scale in the analysis with role functioning as dependent variable was positive. This is in contrast with previous results. In a study with patients with chronic diseases trait anxiety was negatively associated with role physical and role emotional score of the Short-Form 36, a HRQoL questionnaire [54]. Moreover, in colorectal survivors anxiety was significantly associated with lower role functioning over time [55]. To analyse whether this finding was due to multi-collinearity, we correlated the STAI trait scale with the other variables that were associated with role functioning (i.e., CES-D score and ECOG performance status). We observed a strong and positive correlation with CES-D score. This could indicate that the effect of trait anxiety is explained by CES-D score. Second, we observed an, at first glance, unexpected negative direction of the beta of partner status in the analysis with social functioning as dependent variable. However, in a study with advanced-stage cancer patients a similar result was observed [5]. Another study reported also lower social functioning in married/cohabited patients [6]. Moreover, as only weak correlations were observed between partner status and ECOG performance status, CES-D score and age, indications for multi-collinearity were not found.

Some limitations of this study have to be addressed. First, because of the cross-sectional nature of our data, we cannot conclude whether depressive symptoms are a cause of decreased HRQoL and QoL or a consequence, or whether both depressive symptoms and HRQoL and QoL are caused by a third variable. Therefore, ideally, our findings should be cross validated in another study as the observed results may merely describe idiosyncrasies of the data at hand. Second, the relatively small number of patients may have influenced our results. This could have resulted in the non-identification of variables associated with HRQoL and QoL. For this reason, the 10 patients with mesothelioma (i.e., 6.6% of the total patient population) were also used for the analysis. Given that the mesothelioma patients received the same chemotherapy as the other patients, we did not expect differences in terms of number and severity of adverse events between the mesothelioma and the lung cancer patients. Moreover, prognosis in patients with mesothelioma is also limited. Therefore, considering these similarities it was expected that the use of the data of the 10 patients for our analyses would not interfere with the observations of this study. To verify this assumption, the multivariable analyses were rerun without the 10 mesothelioma patients. After Benjamini-Hochberg correction, we observed the same results except that conscientiousness was no longer identified as a predictor of physical health. Third, there is a potential response bias in that possibly the most optimistic of patients or those who had a caregiver who could help with the survey were the ones who returned the questionnaires. Fourth, we related the total CES-D score with HRQoL and QoL. Given that the CES-D contains some items that may demonstrate an overlap with the domains/scales of the WHOQOL-BREF and EORTC QLQ-C30, this could partly explain the observed associations between CES-D score and these domains/scales. However, it was previously demonstrated that just low to moderate correlations exist between the CES-D and the WHOQOL-BREF [56]. Moreover, in the same article results of a Rasch-analysis were reported that demonstrated that just 11 items of the WHOQOL-BREF demonstrated differential item functioning regarding the presence of depression meaning that at a same level of QoL patients with a depression scored these 11 items differently than those without a depression. Removing these 11 items from our analyses would hamper comparing our results with other studies as most studies in cancer do not use an adapted version of the WHOQOL-BREF. In addition, given that the constructs of these questionnaires differ, this also contributes to their utility apart from each other. Fifth, the CES-D also contains some items that demonstrate an overlap with physical symptoms of cancer patients. In potential, this could also partly explain the observed associations between CES-D score and HRQoL and QoL. However, in a study evidence for removing somatic items from the CES-D in cancer patients could not be confirmed (1). In addition, the definition of depressive symptoms includes symptoms like weight loss and fatigue besides symptoms associated with a negative affect. Moreover, to not include the scores of the somatic items in the total CES-D score, would hamper comparing our results with other studies as many studies in cancer exploring depressive symptoms use a total CES-D score.

This study has some strengths too. We are the first to investigate the association between sociodemographic variables, clinical variables, depressive symptoms, and personality traits with both HRQoL and QoL. Moreover, although our sample size was relatively small, we describe results of a prospective study with a homogeneous patient population. Also the application of well-recognized standardized questionnaires, the multi-center prospective design of this study, and the inclusion of patients that resemble clinical practice strengthen our findings.

## Conclusions

In conclusion, our results demonstrated that health care professionals are recommended to have high awareness during consultations for patients with depressive symptoms and those with an ECOG performance status of two or higher at the start of treatment. This is of importance as these factors may indicate low levels of HRQoL and QoL of patients. Moreover, merely assessing HRQoL and QoL and not depressive symptoms or performance status may be insufficient. For instance if psychological health is low, one has to further investigate if this is caused by anxiety or depressive symptoms or another reason given that treatment may differ according to the cause of the low psychological health. Therefore screening for the presence of these two factors before treatment is initiated (e.g., by means of an e-tool that screens for depressive symptoms, consequently reporting performance status during consultations) may be worthwhile. Additional care (e.g., referral to a psychologist, physiotherapist, medication, etc) aimed at improving these factors can then be provided.

## Supplementary information

**Additional file 1.** Online Resource 1.

## Data Availability

The data that support the findings of this study are not publicly available due to them containing information that could compromise research participant privacy/consent but are strictly available from the corresponding author on reasonable request.

## Abbreviations

HRQoL: Health-Related Quality of Life
QoL: Quality of Life
NEO-FFI: Neuroticism-Extraversion-Openness-Five Factor Inventory
CES-D: Center for Epidemiologic Studies Depression
WHOQOL-BREF: World Health Organization Quality of Life-BREF
EORTC QLQ-C30: European Organisation for Research and Treatment of Cancer Quality of Life Questionnaire Core 30
ECOG: Eastern Cooperative Oncology Group
STAI: State-Trait Anxiety Inventory
SD: Standard deviation
IQR: Interquartile range
CI: Confidence interval
n: Number of patients
B: Unstandardized beta
SE: Standard error
β: Standardized beta
R^2^: Explained varriance
