# Supplementary material for: The association of depressive symptoms, personality traits, and sociodemographic factors with health-related quality of life and quality of life in patients with advanced-stage lung cancer: an observational multi-center cohort study
Source: BMC Cancer. 2020 May 18;20:431. doi: 10.1186/s12885-020-06823-3 (PMC7236491; doi:10.1186/s12885-020-06823-3)
Supplement: Supplementary file 1 — Additional file 1. Online Resource 1. [file 12885_2020_6823_MOESM1_ESM.doc]

**Online Resource 1**

**Results of the univariable regression analyses for the WHOQOL-BREF**

| General facet | | | | | | | |
| --- | --- | --- | --- | --- | --- | --- | --- |
| **Independent variables** | **N** | **B** | **SE** | **β** | **P-value** | **95% CI for B** | **R^2^** |
| Age | 142 | -0.031 | 0.015 | -0.168 | 0.046* | -0.061,  -0.001 | 0.028 |
| Gender_a_ | 142 | -0.037 | 0.287 | -0.011 | 0.897 | -0.604, 0.530 | 0.000 |
| Marital status: no partner versus having a partner_b_ | 142 | -0.340 | 0.369 | -0.078 | 0.359 | -1.069, 0.390 | 0.006 |
| Educational level: low versus high_c_ | 137 | 0.092 | 0.340 | 0.023 | 0.788 | -0.581, 0.765 | 0.001 |
| Ethnicity: Caucasian versus other ethnicity_d_ | 142 | -0.885 | 0.583 | -0.127 | 0.131 | -2.037, 0.267 | 0.016 |
| Employment: yes versus having no job_e_ | 142 | 0.840 | 0.318 | 0.218 | 0.009* | 0.211,  1.470 | 0.047 |
| ECOG: 0 to 1 versus 2 or higher_f_ | 140 | -1.246 | 0.471 | -0.220 | 0.009* | -2.177,  -0.315 | 0.048 |
| CES-D | 140 | -0.118 | 0.016 | -0.534 | <0.001* | -0.149,  -0.087 | 0.285 |
| STAI Trait | 139 | -0.075 | 0.026 | -0.236 | 0.005* | -0.128,  -0.023 | 0.056 |
| NEO-FFI neuroticism | 135 | -0.035 | 0.019 | -0.153 | 0.076* | -0.073, 0.004 | 0.024 |
| NEO-FFI extraversion | 130 | 0.049 | 0.022 | 0.196 | 0.025* | 0.006,  0.092 | 0.039 |
| NEO-FFI openness | 131 | 0.014 | 0.025 | 0.049 | 0.579 | -0.036, 0.063 | 0.002 |
| NEO-FFI agreeableness | 136 | 0.017 | 0.029 | 0.051 | 0.555 | -0.040, 0.073 | 0.003 |
| NEO-FFI conscientiousness | 131 | 0.050 | 0.025 | 0.172 | 0.049* | 0.000,  0.100 | 0.030 |
|  |  |  |  |  |  |  |  |
|  | Physical health | | | | | | |
| **Independent variables** | **N** | **B** | **SE** | **β** | **P-value** | **95% CI for B** | **R^2^** |
| Age | 145 | 0.014 | 0.028 | 0.042 | 0.614 | -0.042, 0.071 | 0.002 |
| Gender_a_ | 145 | -0.757 | 0.518 | -0.121 | 0.146 | -1.780, 0.266 | 0.008 |
| Marital status: no partner versus having a partner_b_ | 145 | -0.805 | 0.664 | -0.101 | 0.228 | -2.118, 0.509 | 0.010 |
| Educational level: low versus high_c_ | 140 | 0.220 | 0.634 | 0.029 | 0.730 | -1.034, 1.473 | 0.001 |
| Ethnicity: Caucasian versus other ethnicity_d_ | 145 | -0.292 | 1.077 | -0.023 | 0.787 | -2.421, 1.837 | 0.001 |
| Employment: yes versus having no job_e_ | 145 | 1.446 | 0.584 | 0.203 | 0.014* | 0.292,  2.600 | 0.041 |
| ECOG: 0 to 1 versus 2 or higher_f_ | 143 | -3.167 | 0.845 | -0.301 | <0.001* | -4.837,  -1.498 | 0.091 |
| CES-D | 143 | -0.234 | 0.028 | -0.575 | <0.001* | -0.289,  -0.179 | 0.331 |
| STAI Trait | 142 | -0.211 | 0.047 | -0.356 | <0.001* | -0.303,  -0.118 | 0.127 |
| NEO-FFI neuroticism | 137 | -0.124 | 0.035 | -0.296 | <0.001* | -0.193,  -0.056 | 0.087 |
| NEO-FFI extraversion | 133 | 0.099 | 0.040 | 0.210 | 0.015* | 0.019,  0.178 | 0.044 |
| NEO-FFI openness | 134 | -0.071 | 0.046 | -0.132 | 0.128 | -0.163, 0.021 | 0.017 |
| NEO-FFI agreeableness | 139 | 0.112 | 0.053 | 0.177 | 0.037* | 0.007,  0.216 | 0.031 |
| NEO-FFI conscientiousness | 134 | 0.162 | 0.046 | 0.291 | 0.001* | 0.070,  0.254 | 0.084 |
|  |  |  |  |  |  |  |  |
|  | Psychological health | | | | | | |
| **Independent variables** | **N** | **B** | **SE** | **β** | **P-value** | **95% CI for B** | **R^2^** |
| Age | 145 | 0.008 | 0.022 | 0.032 | 0.704 | -0.035, 0.051 | 0.001 |
| Gender_a_ | 145 | -0.357 | 0.397 | -0.075 | 0.370 | -1.142, 0.427 | 0.006 |
| Marital status: no partner versus having a partner_b_ | 145 | 0.236 | 0.510 | 0.039 | 0.644 | -0.771, 1.243 | 0.002 |
| Educational level: low versus high_c_ | 140 | 0.481 | 0.473 | 0.086 | 0.310 | -0.453, 1.416 | 0.007 |
| Ethnicity: Caucasian versus other ethnicity_d_ | 145 | -1.011 | 0.818 | -0.103 | 0.219 | -2.629, 0.606 | 0.011 |
| Employment: yes versus having no job_e_ | 145 | 0.521 | 0.453 | 0.096 | 0.252 | -0.375, 1.417 | 0.009 |
| ECOG: 0 to 1 versus 2 or higher_f_ | 143 | -1.582 | 0.663 | -0.197 | 0.018* | -2892,  -0.272 | 0.039 |
| CES-D | 143 | -0.201 | 0.020 | -0.653 | <0.001* | -0.240,  -0.162 | 0.427 |
| STAI Trait | 142 | -0.233 | 0.032 | -0.518 | <0.001* | -0.297,  -0.168 | 0.268 |
| NEO-FFI neuroticism | 137 | -0.158 | 0.024 | -0.494 | <0.001* | -0.205,  -0.110 | 0.244 |
| NEO-FFI extraversion | 133 | 0.101 | 0.030 | 0.278 | 0.001* | 0.041,  0.161 | 0.078 |
| NEO-FFI openness | 134 | 0.001 | 0.035 | 0.002 | 0.983 | -0.069, 0.070 | 0.000 |
| NEO-FFI agreeableness | 139 | 0.076 | 0.039 | 0.163 | 0.056* | -0.002, 0.154 | 0.027 |
| NEO-FFI conscientiousness | 134 | 0.129 | 0.034 | 0.314 | <0.001* | 0.062,  0.197 | 0.098 |
|  |  |  |  |  |  |  |  |
|  | Social relationships | | | | | | |
| **Independent variables** | **N** | **B** | **SE** | **β** | **P-value** | **95% CI for B** | **R^2^** |
| Age | 145 | 0.20 | 0.023 | 0.074 | 0.377 | -0.025, 0.066 | 0.005 |
| Gender_a_ | 145 | 0.938 | 0.417 | 0.185 | 0.026* | 0.115,  1.762 | 0.034 |
| Marital status: no partner versus having a partner_b_ | 145 | 1.105 | 0.535 | 0.170 | 0.041* | 0.047,  2.162 | 0.029 |
| Educational level: low versus high_c_ | 140 | 0.658 | 0.500 | 0.111 | 0.190 | -0.330, 1.646 | 0.012 |
| Ethnicity: Caucasian versus other ethnicity_d_ | 145 | 0.511 | 0.875 | 0.049 | 0.560 | -1.219, 2.240 | 0.002 |
| Employment: yes versus having no job_e_ | 145 | -0.106 | 0.485 | -0.018 | 0.828 | -1.064, 0.852 | 0.000 |
| ECOG: 0 to 1 versus 2 or higher_f_ | 143 | -1.786 | 0.697 | -0.211 | 0.011* | -3.163,  -0.409 | 0.045 |
| CES-D | 143 | -0.056 | 0.027 | -0.168 | 0.044* | -0.110,  -0.001 | 0.028 |
| STAI Trait | 142 | -0.108 | 0.040 | -0.225 | 0.007* | -0.186,  -0.030 | 0.051 |
| NEO-FFI neuroticism | 137 | -0.078 | 0.028 | -0.230 | 0.007* | -0.133,  -0.022 | 0.053 |
| NEO-FFI extraversion | 133 | 0.080 | 0.032 | 0.216 | 0.012* | 0.018,  0.143 | 0.047 |
| NEO-FFI openness | 134 | 0.016 | 0.036 | 0.039 | 0.658 | -0.056, 0.088 | 0.001 |
| NEO-FFI agreeableness | 139 | 0.068 | 0.042 | 0.139 | 0.103 | -0.014, 0.150 | 0.019 |
| NEO-FFI conscientiousness | 134 | 0.104 | 0.037 | 0.238 | 0.006* | 0.031,  0.177 | 0.057 |
|  |  |  |  |  |  |  |  |
|  | Environment | | | | | | |
| **Independent variables** | **N** | **B** | **SE** | **β** | **P-value** | **95% CI for B** | **R^2^** |
| Age | 145 | 0.017 | 0.020 | 0.069 | 0.409 | -0.023, 0.057 | 0.005 |
| Gender_a_ | 145 | 0.340 | 0.369 | 0.077 | 0.358 | -0.390, 1.071 | 0.006 |
| Marital status: no partner versus having a partner_b_ | 145 | 0.450 | 0.473 | 0.079 | 0.343 | -0.485, 1.385 | 0.006 |
| Educational level: low versus high_c_ | 140 | 0.903 | 0.445 | 0.170 | 0.044* | 0.023,  1.783 | 0.029 |
| Ethnicity: Caucasian versus other ethnicity_d_ | 145 | 0.092 | 0.766 | 0.010 | 0.905 | -1.422, 1.605 | 0.000 |
| Employment: yes versus having no job_e_ | 145 | 0.381 | 0.423 | 0.075 | 0.369 | -0.455, 1.216 | 0.006 |
| ECOG: 0 to 1 versus 2 or higher_f_ | 143 | -0.918 | 0.624 | -0.123 | 0.143 | -2.152, 0.315 | 0.015 |
| CES-D | 143 | -0.134 | 0.022 | -0.465 | <0.001* | -0.177,  -0.092 | 0.216 |
| STAI Trait | 142 | -0.221 | 0.030 | -0.522 | <0.001* | -0.281,  -0.161 | 0.273 |
| NEO-FFI neuroticism | 137 | -0.116 | 0.024 | -0.389 | <0.001* | -0.162,  -0.069 | 0.152 |
| NEO-FFI extraversion | 133 | 0.069 | 0.028 | 0.209 | 0.016* | 0.013,  0.125 | 0.044 |
| NEO-FFI openness | 134 | -0.022 | 0.033 | -0.059 | 0.500 | -0.088, 0.043 | 0.003 |
| NEO-FFI agreeableness | 139 | 0.086 | 0.036 | 0.198 | 0.020* | 0.014,  0.158 | 0.039 |
| NEO-FFI conscientiousness | 134 | 0.115 | 0.033 | 0.295 | 0.001* | 0.051,  0.180 | 0.087 |

*P-values of p≤ 0.10

_a_Male is reference

_b_No partner is reference

_c_Low educational level is reference

_d_Other ethnicity is reference

_e_No job is reference

_f_0 to 1 is reference

CES-D score, STAI trait score and NEO-FFI scale scores represent continuous variables

Abbreviations: N, number of patients; B, unstandardized beta; SE, standard error; β, standardized beta**,**  CI, confidence interval; R2; WHOQOL-BREF, World Health Organization Quality of Life-BREF questionnaire; ECOG, Eastern Cooperative Oncology Group; CES-D, Center for Epidemiologic Studies Depression Scale; STAI, State Trait Anxiety Inventory; NEO-FFI, Neuroticism-Extraversion-Openness Five-Factor Inventory

Results of the univariable regression analyses for the EORTC QLQ-C30

|  | Global Health Status/QoL | | | | | | |
| --- | --- | --- | --- | --- | --- | --- | --- |
| **Independent variables** | **n** | **B** | **SE** | **β** | **P-value** | **95% CI for B** | **R^2^** |
| Age | 142 | -0.144 | 0.235 | -0.052 | 0.540 | -0.608, 0.320 | 0.003 |
| Gender_a_ | 142 | -5.815 | 4.275 | -0.114 | 0.176 | -14.267, 2.638 | 0.013 |
| Marital status: no partner versus having a partner_b_ | 142 | -2.813 | 5.545 | -0.043 | 0.613 | -13.775, 8.149 | 0.002 |
| Educational level: low versus high_c_ | 137 | -2.837 | 5.135 | -0.047 | 0.582 | -12.994, 7.319 | 0.002 |
| Ethnicity: Caucasian versus other ethnicity_d_ | 142 | -5.799 | 8.796 | -0.056 | 0.511 | -23.189, 11.590 | 0.003 |
| Employment: yes versus having no job_e_ | 142 | 14.893 | 4.725 | 0.257 | 0.002* | 5.551, 24.234 | 0.066 |
| ECOG: 0 to 1 versus 2 or higher_f_ | 140 | -17.063 | 7.104 | -0.200 | 0.018* | -31.111,  -3.016 | 0.040 |
| CES-D | 142 | -1.940 | 0.225 | -0.589 | <0.001* | -2.385,  -1.495 | 0.346 |
| STAI Trait | 139 | -1.246 | 0.396 | -0.260 | 0.002* | -2.029,  -0.463 | 0.067 |
| NEO-FFI neuroticism | 134 | -0.938 | 0.292 | -0.269 | 0.002* | -1.516,  -0.360 | 0.072 |
| NEO-FFI extraversion | 130 | 0.687 | 0.333 | 0.179 | 0.041* | 0.028,  1.347 | 0.032 |
| NEO-FFI openness | 132 | -0.196 | 0.377 | -0.045 | 0.605 | -0.942, 0.551 | 0.002 |
| NEO-FFI agreeableness | 136 | 0.757 | 0.423 | 0.153 | 0.076* | -0.079, 1.594 | 0.023 |
| NEO-FFI conscientiousness | 131 | 1.084 | 0.383 | 0.242 | 0.005* | 0.327,  1.841 | 0.059 |
|  |  |  |  |  |  |  |  |
|  | Physical functioning | | | | | | |
| **Independent variables** | **n** | **B** | **SE** | **β** | **P-value** | **95% CI for B** | **R^2^** |
| Age | 150 | -0.006 | 0.218 | -0.002 | 0.978 | -0.436, 0.424 | 0.000 |
| Gender_a_ | 150 | -10.493 | 3.869 | -0.218 | 0.007* | -18.138,  -2.847 | 0.047 |
| Marital status: no partner versus having a partner_b_ | 150 | -4.692 | 5.050 | -0.076 | 0.354 | -14.671, 5.288 | 0.006 |
| Educational level: low versus high_c_ | 145 | -0.347 | 4.858 | -0.006 | 0.943 | -9.950, 9.257 | 0.000 |
| Ethnicity: Caucasian versus other ethnicity_d_ | 150 | -0.063 | 8.309 | -0.001 | 0.994 | -16.484, 16.357 | 0.000 |
| Employment: yes versus having no job_e_ | 150 | 13.603 | 4.397 | 0.246 | 0.002* | 4.913, 22.293 | 0.061 |
| ECOG: 0 to 1 versus 2 or higher_f_ | 148 | -25.686 | 6.490 | -0.311 | <0.001* | -38.512,  -12.860 | 0.097 |
| CES-D | 148 | -1.516 | 0.228 | -0.482 | <0.001* | -1.967,  -1.065 | 0.232 |
| STAI Trait | 147 | -0.961 | 0.374 | -0.209 | 0.011* | -1.701,  -0.222 | 0.044 |
| NEO-FFI neuroticism | 137 | -0.511 | 0.266 | -0.163 | 0.057* | -1.036, 0.015 | 0.027 |
| NEO-FFI extraversion | 133 | 0.647 | 0.310 | 0.179 | 0.039* | 0.034,  1.259 | 0.032 |
| NEO-FFI openness | 134 | -0.456 | 0.346 | -0.114 | 0.189 | -1.140, 0.228 | 0.013 |
| NEO-FFI agreeableness | 139 | 0.682 | 0.397 | 0.145 | 0.088* | -0.103, 1.467 | 0.021 |
| NEO-FFI conscientiousness | 134 | 1.015 | 0.352 | 0.243 | 0.005* | 0.318,  1.712 | 0.059 |
|  |  |  |  |  |  |  |  |
|  | Role functioning | | | | | | |
| **Independent variables** | **n** | **B** | **SE** | **β** | **P-value** | **95% CI for B** | **R^2^** |
| Age | 149 | 0.162 | 0.297 | 0.045 | 0.585 | -0.424, 0.748 | 0.002 |
| Gender_a_ | 149 | -12.170 | 5.322 | -0.185 | 0.024* | -22.688,  -1.653 | 0.034 |
| Marital status: no partner versus having a partner_b_ | 149 | -3.193 | 6.900 | -0.038 | 0.644 | -16.829, 10.444 | 0.001 |
| Educational level: low versus high_c_ | 144 | -0.595 | 6.592 | -0.008 | 0.928 | -13.626, 12.436 | 0.000 |
| Ethnicity: Caucasian versus other ethnicity_d_ | 149 | -2.407 | 11.322 | -0.018 | 0.832 | -24.782, 19.967 | 0.000 |
| Employment: yes versus having no job_e_ | 149 | 10.167 | 6.132 | 0.135 | 0.099* | -1.951, 22.285 | 0.018 |
| ECOG: 0 to 1 versus 2 or higher_f_ | 147 | -35.526 | 8.769 | -0.319 | <0.001* | -52.858,  -18.194 | 0.102 |
| CES-D | 148 | -2.263 | 0.304 | -0.525 | <0.001* | -2.863,  -1.663 | 0.276 |
| STAI Trait | 146 | -0.938 | 0.511 | -0.151 | 0.069* | -1.949, 0.072 | 0.023 |
| NEO-FFI neuroticism | 136 | -0.940 | 0.365 | -0.217 | 0.011* | -1.662,  -0.218 | 0.047 |
| NEO-FFI extraversion | 132 | 0.664 | 0.421 | 0.137 | 0.117 | -0.169, 1.498 | 0.019 |
| NEO-FFI openness | 134 | -0.852 | 0.473 | -0.155 | 0.074* | -1.788, 0.084 | 0.024 |
| NEO-FFI agreeableness | 138 | 0.864 | 0.546 | 0.135 | 0.116 | -0.215, 1.944 | 0.018 |
| NEO-FFI conscientiousness | 133 | 1.067 | 0.488 | 0.187 | 0.031* | 0.101,  2.033 | 0.035 |
|  |  |  |  |  |  |  |  |
|  | Emotional functioning | | | | | | |
| **Independent variables** | **n** | **B** | **SE** | **β** | **P-value** | **95% CI for B** | **R^2^** |
| Age | 142 | -0.193 | 0.221 | -0.074 | 0.382 | -0.630, 0.243 | 0.005 |
| Gender_a_ | 142 | -1.883 | 4.049 | -0.039 | 0.643 | -9.888, 6.122 | 0.002 |
| Marital status: no partner versus having a partner_b_ | 142 | -8.175 | 5.180 | -0.132 | 0.117 | -18.416, 2.066 | 0.017 |
| Educational level: low versus high_c_ | 137 | 2.582 | 4.858 | 0.046 | 0.596 | -7.027, 12.190 | 0.002 |
| Ethnicity: Caucasian versus other ethnicity_d_ | 142 | -3.328 | 8.290 | -0.034 | 0.689 | -19.718, 13.062 | 0.001 |
| Employment: yes versus having no job_e_ | 142 | 6.517 | 4.571 | 0.120 | 0.156 | -2.521, 15.554 | 0.014 |
| ECOG: 0 to 1 versus 2 or higher_f_ | 140 | -10.053 | 6.678 | -0.127 | 0.135 | -23.257, 3.152 | 0.016 |
| CES-D | 142 | -2.438 | 0.162 | -0.786 | <0.001* | -2.759,  -2.117 | 0.617 |
| STAI Trait | 139 | -2.713 | 0.312 | -0.597 | <0.001* | -3.330,  -2.096 | 0.356 |
| NEO-FFI neuroticism | 134 | -1.727 | 0.244 | -0.525 | <0.001* | -2.208,  -1.245 | 0.276 |
| NEO-FFI extraversion | 130 | 0.725 | 0.309 | 0.203 | 0.020* | 0.114,  1.335 | 0.041 |
| NEO-FFI openness | 132 | 0.240 | 0.360 | 0.058 | 0.507 | -0.474, 0.953 | 0.003 |
| NEO-FFI agreeableness | 136 | 1.136 | 0.397 | 0.240 | 0.005* | 0.351,  1.921 | 0.058 |
| NEO-FFI conscientiousness | 131 | 0.769 | 0.369 | 0.181 | 0.039* | 0.039,  1.499 | 0.033 |
|  |  |  |  |  |  |  |  |
|  | Cognitive functioning | | | | | | |
| **Independent variables** | **n** | **B** | **SE** | **β** | **P-value** | **95% CI for B** | **R^2^** |
| Age | 142 | 0.145 | 0.213 | 0.058 | 0.496 | -0.275, 0.566 | 0.003 |
| Gender_a_ | 142 | -7.509 | 3.851 | -0.163 | 0.053* | -15.124, 0.105 | 0.026 |
| Marital status: no partner versus having a partner_b_ | 142 | -0.597 | 5.033 | -0.010 | 0.906 | -10.548, 9.355 | 0.000 |
| Educational level: low versus high_c_ | 137 | 11.602 | 4.554 | 0.214 | 0.012* | 2.596, 20.608 | 0.046 |
| Ethnicity: Caucasian versus other ethnicity_d_ | 142 | 0.696 | 7.990 | 0.007 | 0.931 | -15.100, 16.493 | 0.000 |
| Employment: yes versus having no job_e_ | 142 | 7.782 | 4.386 | 0.148 | 0.078* | -0.890, 16.454 | 0.022 |
| ECOG: 0 to 1 versus 2 or higher_f_ | 140 | 0.926 | 6.565 | 0.012 | 0.888 | -12.054, 13.906 | 0.000 |
| CES-D | 142 | -1.720 | 0.207 | -0.575 | <0.001* | -2.128,  -1.311 | 0.331 |
| STAI Trait | 139 | -1.495 | 0.352 | -0.341 | <0.001* | -2.192,  -0.799 | 0.116 |
| NEO-FFI neuroticism | 134 | -0.867 | 0.263 | -0.276 | 0.001* | -1.387,  -0.347 | 0.076 |
| NEO-FFI extraversion | 130 | 0.057 | 0.312 | 0.016 | 0.856 | -0.561, 0.675 | 0.000 |
| NEO-FFI openness | 132 | -0.076 | 0.341 | -0.020 | 0.824 | -0.750, 0.598 | 0.000 |
| NEO-FFI agreeableness | 136 | 0.647 | 0.395 | 0.140 | 0.104 | -0.134, 1.427 | 0.020 |
| NEO-FFI conscientiousness | 131 | 0.530 | 0.350 | 0.132 | 0.132 | -0.162, 1.223 | 0.017 |
|  |  |  |  |  |  |  |  |
|  | Social functioning | | | | | | |
| **Independent variables** | **n** | **B** | **SE** | **β** | **P-value** | **95% CI for B** | **R^2^** |
| Age | 142 | 0.353 | 0.248 | 0.120 | 0.156 | -0.136, 0.843 | 0.014 |
| Gender_a_ | 142 | -5.688 | 4.541 | -0.105 | 0.212 | -14.665, 3.289 | 0.011 |
| Marital status: no partner versus having a partner_b_ | 142 | -11.373 | 5.809 | -0.163 | 0.052* | -22.858, 0.113 | 0.027 |
| Educational level: low versus high_c_ | 137 | 1.558 | 5.487 | 0.024 | 0.777 | -9.294, 12.409 | 0.001 |
| Ethnicity: Caucasian versus other ethnicity_d_ | 142 | -8.702 | 9.318 | -0.079 | 0.352 | -27.124, 9.720 | 0.006 |
| Employment: yes versus having no job_e_ | 142 | 11.158 | 5.102 | 0.182 | 0.030* | 1.072, 21.245 | 0.033 |
| ECOG: 0 to 1 versus 2 or higher_f_ | 140 | -19.841 | 7.506 | -0.220 | 0.009* | -34.684,  -4.999 | 0.048 |
| CES-D | 142 | -1.765 | 0.255 | -0.505 | <0.001* | -2.269,  -1.260 | 0.255 |
| STAI Trait | 139 | -1.435 | 0.421 | -0.280 | 0.001* | -2.267,  -0.604 | 0.078 |
| NEO-FFI neuroticism | 134 | -0.835 | 0.306 | -0.231 | 0.007* | -1.440,  -0.230 | 0.053 |
| NEO-FFI extraversion | 130 | 0.798 | 0.355 | 0.195 | 0.026* | 0.096,  1.500 | 0.038 |
| NEO-FFI openness | 132 | -0.305 | 0.400 | -0.067 | 0.447 | -1.098, 0.487 | 0.004 |
| NEO-FFI agreeableness | 136 | 0.789 | 0.457 | 0.148 | 0.086* | -0.114, 1.692 | 0.022 |
| NEO-FFI conscientiousness | 131 | 1.371 | 0.396 | 0.292 | 0.001* | 0.588,  2.155 | 0.085 |
|  |  |  |  |  |  |  |  |

*P-values of ≤ 0.10

_a_Male is reference

_b_No partner is reference

_c_Low educational level is reference

_d_Other ethnicity is reference

_e_No job is reference

_f_0 to 1 is reference

CES-D, STAI trait, and NEO-FFI scale scores represent continuous variables

Abbreviations: n, number of patients; B, unstandardized beta; SE, standard error; β, standardized beta**,**  CI, confidence interval; R2; EORTC QLQ-C30, European Organization for Research and Treatment of Cancer Quality of Life Questionnaire Core 30; ECOG, Eastern Cooperative Oncology Group; CES-D, Center for Epidemiologic Studies Depression Scale; STAI, State Trait Anxiety Inventory; NEO-FFI, Neuroticism-Extraversion-Openness Five-Factor Inventory
